# In-Home Free-Living Gait Monitoring Using a Single Lumbar IMU Reveals Nonlinear Complexity Changes During Intensive Stroke Rehabilitation

**DOI:** 10.3390/s26206467

**Published:** 2026-10-13

**Authors:** Rahul Soangra, Susan V. Duff, Alison McKenzie

**Affiliations:** 1 Crean College of Health & Behavioral Sciences, Chapman University, Irvine, CA 92618, USA; duff@chapman.edu (S.V.D.); amckenzi@chapman.edu (A.M.); 2 Fowler School of Engineering, Chapman University, Irvine, CA 92618, USA

**Keywords:** in-home assessment, rehab assessment, wearable gait analysis, stroke boot camp, stroke rehabilitation

## Abstract

Conventional clinical assessments of stroke rehabilitation are limited to supervised environments and fail to capture continuous real-world motor behavior. This proof-of-concept pilot study (case series, n = 3) evaluated the feasibility and longitudinal sensitivity of wearable IMU-derived digital biomarkers of three individuals with chronic stroke across Chapman University’s Stroke Boot Camp (SBC), a two-week intensive interdisciplinary rehabilitation program. A lower-back-mounted DynaPort McRoberts IMU provided continuous multi-day free-living monitoring across five sessions that spanned one week pre-intervention through to three months post-intervention. A validated Python-based pipeline implemented Mobilise-D gait sequence detection, wavelet-based heel strike and toe-off identification, and extraction of linear spatiotemporal parameters alongside a 24-feature nonlinear biomarker panel, which included sample entropy, multi-scale entropy variants, Lyapunov exponents, and recurrence quantification analysis metrics. A standardized clinical battery (FMA, BBS, FGA, 10 MWT, 6 MWT) was administered at each session. All three participants exhibited a 52–62% increase in Wolf’s Lyapunov exponent during SBC Week 2, which was most consistent across participants. Participant-specific entropy responses were heterogeneous, with SampEn and RCMSE showing greater longitudinal sensitivity than conventional spatiotemporal metrics. These results demonstrate that a single lumbar IMU, paired with a nonlinear analytics pipeline, can detect gait reorganization during intensive stroke rehabilitation imperceptible to standard clinical measures, providing a reproducible methodological foundation for larger controlled trials.

## 1. Introduction

Stroke remains a leading cause of long-term disability worldwide, with an estimated 12.2 million new cases annually and more than 101 million individuals living with its sequelae globally [1]. Chronic stroke commonly manifests as persistent deficits in gait, balance, upper-extremity function, and postural control, each of which compounds sedentary behavior and fall risk and diminishes quality of life [2]. Intensive, community-based rehabilitation programs, including task-specific movement training, balance challenges, and aerobic conditioning, have demonstrated efficacy in improving neuromotor function and daily mobility in the chronic phase [3,4]. However, accurately quantifying the magnitude, trajectory, and durability of these improvements remains a fundamental challenge for the field. Conventional clinical assessments, such as the 10-Meter Walk Test, Berg Balance Scale, and Fugl-Meyer Assessment, provide valuable snapshot evaluations but are inherently constrained to controlled laboratory or clinic environments, are susceptible to assessor and day-of-testing variability, and cannot capture the nuanced, continuous dynamics of real-world motor behavior across a rehabilitation continuum [5,6].

The emergence of wearable inertial measurement units (IMUs) as digital biomarker platforms offers a fundamentally different paradigm for rehabilitation monitoring. Miniaturized accelerometers and gyroscopes enable continuous, unobtrusive capture of full-day ambulatory behavior in naturalistic home and community environments, yielding ecologically valid mobility data that clinical snapshots cannot replicate [7,8,9]. Growing evidence highlights that movement behavior in laboratory settings frequently diverges from habitual daily-life patterns; gait characteristics, such as speed, cadence, step regularity, and locomotor complexity, differ systematically between supervised and free-living conditions due to attentional demands, environmental constraints, and motivational context [10,11]. Critically, individuals with stroke often demonstrate reduced and highly variable community ambulation, including shorter and irregular walking bouts and degraded locomotor complexity that are not fully captured by single-session clinical evaluations [12,13]. This complex heterogeneity during movements among stroke individuals extends to underlying control strategies; for example, causal-network modeling of free-living stroke gait has revealed a distinct individualized gait control phenotype rather than a single impairment continuum [14]. Thus, multi-day wearable monitoring offers a more ecologically valid measure of mobility by capturing gait across the varied environmental and task demands of daily life, such as navigating constrained spaces, moving around furniture or crowds, changing pace, carrying objects, and walking under dual-task conditions. This continuous monitoring also enables each participant to be compared against their own baseline, reducing between-subject variability and improving sensitivity to true functional change [15,16]. Beyond inertial sensing, emerging wearable technologies are expanding the range of digital biomarkers. For example, photonic smart wristbands using all-polymer sensing units can continuously monitor respiratory rate, heart rate, and blood pressure while providing accurate biometric identification [17]. These advances highlight the broader multimodal wearable-health ecosystem that complements IMU-based gait analytics. Extracting clinically meaningful digital biomarkers from multi-day IMU recordings requires a rigorous, multi-stage signal-processing pipeline. Raw tri-axial accelerometry from a lower-back sensor encodes the ensemble of daily activities as a complex, non-stationary time series that must be systematically segmented, filtered, and decomposed into walking-specific epochs before parametric characterization is possible. Gait sequence detection (GSD) algorithms, validated through the Mobilise-D consortium for both healthy and clinical populations, separate ambulatory bouts from non-walking activities using either sine-template convolution with sliding-window variance thresholding (GSDA; targeting healthy gait) or a multi-stage continuous wavelet transform (CWT) and Hilbert envelope-adaptive thresholding approach (GSDB; targeting impaired, slower gait patterns) [18,19,20]. Within detected gait sequences, initial contact or heel strike (HS) and final contact or toe-off (TO) events are identified from the CWT-transformed vertical velocity signal, providing the step-level temporal resolution necessary for bilateral spatiotemporal gait parameter extraction [21]. Linear gait parameters, including gait cycle time, double-support time, single-support time, swing time, and cadence, are derived from alternating heel strike and toe-off sequences and capture the temporal structure and inter-limb symmetry of locomotion [22]. These conventional metrics are complemented by nonlinear dynamical measures that characterize the complexity, regularity, and local stability of locomotor patterns in ways that linear statistics cannot. Approximate entropy (ApEn) and sample entropy (SampEn) quantify stride-to-stride signal regularity; permutation entropy (PermEn) and symbolic entropy (SymEn) capture coarse-grained distributional variability; multi-scale entropy variants (MSE, CMSE, RCMSE) assess complexity across temporal scales spanning tens to hundreds of milliseconds; and Lyapunov exponents (Rosenstein and Wolf methods), together with recurrence quantification analysis (RQA) metrics, quantify orbital instability and deterministic structure within the locomotor attractor [23,24]. Together, these linear and nonlinear biomarkers constitute a multidimensional fingerprint of gait quality that encodes information beyond what individual clinical scales can resolve. This mechanistic transparency distinguishes the present nonlinear approach from deep-learning gait-recognition models (e.g., convolutional neural networks) [25]; while CNN-based pipelines excel at classifying gait sequences in daily-life data, they operate largely as black boxes, whereas metrics such as Wolf’s Lyapunov exponent carry an explicit dynamical-systems interpretation as a marker of local stability and motor adaptation.

A key advantage of sensor-derived digital biomarkers in rehabilitation monitoring is their ability to detect subtle, subclinical improvements that may precede, or be more sensitive than, changes identified by conventional assessments. Intensive stroke rehabilitation may induce neuroplastic changes in motor cortex organization, corticospinal connectivity, and cerebellar–basal ganglia networks, which can initially present as small but consistent improvements in motor timing, gait regularity, and postural adaptation [26,27]. A critical next step is to determine whether these subtle sensor-detected changes translate into meaningful functional gains, such as improved community mobility, greater independence in daily activities, reduced fall risk, and enhanced participation. Nonlinear gait metrics may be particularly sensitive to early rehabilitation-related improvements because these reflect the dynamic process of motor relearning. Consistent with motor learning theory, individuals may initially demonstrate greater variability and movement errors as they explore new gait strategies, followed by more stable and efficient movement patterns with practice. Thus, increases in locomotor complexity, reflected by higher entropy measures, alongside improved local dynamic stability, reflected by lower short-term Lyapunov exponents and greater RQA determinism, may represent a shift toward more adaptive and skilled gait control in neurological populations [28,29]. Crucially, longitudinal within-subject analysis across multiple time points amplifies this sensitivity; when each participant serves as their own control across a rehabilitation trajectory, spanning *pre-intervention*, intervention, *post-intervention*, and *follow-up windows*, trial-to-trial noise and inter-individual motor variability are substantially attenuated, increasing the statistical power to detect functionally meaningful change, even in small pilot cohorts [30,31]. In addition to trunk-based gait metrics, quantifying upper- and lower-extremity-use ratios from wrist- and ankle-mounted IMUs is important for tracking rehabilitation progress after stroke, as these sensors capture asymmetries in functional arm use, stepping, and limb coordination during daily activities [32,33,34].

Despite the promise of wearable digital biomarkers for stroke rehabilitation monitoring, the field still lacks standardized, scalable workflows for processing multi-day real-world IMU recordings and quantifying longitudinal change with sufficient rigor. Heterogeneity in preprocessing, gait sequence detection, feature extraction, and longitudinal aggregation limits reproducibility and cross-study comparability [20,35]. Thus, validated pipelines that integrate automated data ingestion, population-appropriate gait sequence detection, step-event identification, and comprehensive linear and nonlinear biomarker extraction are needed for longitudinal rehabilitation studies [7,36]. The Stroke Boot Camp at Chapman University provides an ideal setting to address this gap because its intensive two-week interdisciplinary structure and repeated assessments enable within-subject characterization of recovery trajectories across pre-intervention, intervention, post-intervention, and follow-up time points. Accordingly, this pilot study was designed to evaluate the feasibility and longitudinal sensitivity of wearable sensor-derived digital biomarkers across this structured rehabilitation program. Given the small pilot cohort, the primary contribution of this work is methodological: to demonstrate a reproducible longitudinal IMU analytics framework and identify digital biomarkers most sensitive to subtle functional change during intensive stroke rehabilitation.

## 2. Materials and Methods

### 2.1. Study Design

This study employed a pilot group with a longitudinal observational design to characterize wearable sensor-derived digital biomarkers across the trajectory of an intensive community-based stroke rehabilitation program. Five data collection sessions were conducted per participant, spanning a window from one week before the intervention through to three months post-intervention. The primary objective was methodological: to demonstrate the feasibility and sensitivity of a multi-sensor signal analysis pipeline for detecting within-subject changes in free-living gait and movement behavior across a structured rehabilitation continuum. Consistent with a methods-oriented pilot design, no control group was included, and inferential statistics regarding intervention efficacy were not the primary focus. The study was reviewed and approved by the Institutional Review Board at Chapman University, and all participants provided written informed consent prior to enrollment.

### 2.2. Participants

Three participants were recruited from Chapman University’s Stroke Boot Camp (SBC). SBC is a twice-yearly community-based intensive interdisciplinary rehabilitation program for individuals with chronic stroke. Participants included two males and one female, with a mean age of 48.3 ± 19.6 years. All participants were in the chronic phase of stroke recovery, with times since stroke ranging from 46 to 62 months. The mean time since stroke was 55.0 ± 8.2 months. Two participants experienced a right-sided stroke resulting in left-sided hemiparesis, whereas one participant experienced a left-sided stroke resulting in right-sided hemiparesis. All participants were right-hand dominant. Participants had a mean education level of 16.7 ± 1.2 years, a mean height of 173.5 ± 11.9 cm, and a mean body weight of 92.1 ± 19.3 kg. Eligibility for this study required a diagnosis of unilateral ischemic or hemorrhagic chronic (i.e., >6 months) stroke with residual motor deficits; the ability to ambulate at least 2 m, with or without an assistive device, with a maximum of 2 people assisting; the ability to follow 2-step commands; and the absence of concurrent neurological conditions that would confound gait analysis.

### 2.3. Study Protocol

Each participant completed five structured data collection sessions corresponding to distinct phases of the rehabilitation continuum: pre-intervention baseline, acute intervention onset, acute intervention end, early follow-up, and extended follow-up. The session schedules and associated data collection activities are summarized in Table 1.

*Session 1—Baseline (One Week Pre-SBC):* At the initial session, after providing consent, participants underwent a full battery of clinical assessments (FMA, BBS, FGA, 10 MWT, 6 MWT, BBT, SIS, COPM, GD) in the laboratory. Following assessment, participants were fitted with both sensor systems. The DynaPort sensor (McRoberts B.V., The Hague, The Netherlands) was worn continuously on the lower back for 3 days to capture habitual free-living activity before SBC. Originally developed for monitoring gait in people with Parkinson’s Disease, Ambulatory Parkinson’s Disease Monitoring (APDM) Opal sensors (https://clario.com/solutions/precision-motion-for-research/, accessed on 20 September 2026) were worn on both wrists and ankles for one 7–8 h day within the same week to capture upper- and lower-extremity activities during daily life. Participants received standardized instructions for wear, removal, and charging. *Session 2—First Day of SBC*: Clinical assessments were repeated at the start of SBC. Dynaport monitoring began continuously during this session and continued throughout the 2-week SBC period and into the 1-month follow-up. *Session 3—Last Day of SBC (Approximately Two Weeks Post-Session 2):* Clinical assessments were repeated at the end of SBC. APDM sensors were worn for a second single-day session to capture movement at program completion. Dynaport monitoring continued uninterrupted for 3 days. *Session 4—One Month Post-SBC*: One month following the conclusion of SBC, participants returned to the laboratory for a fourth clinical assessment session. The APDM sensors were worn for a single 7–8 h instrumented day coinciding with this visit. The Dynaport sensor was worn continuously on the lower back for 3 days to capture habitual free-living activity. *Session 5—Three Months Post-SBC:* At the final follow-up, participants completed the full clinical assessment battery. APDM sensors were worn for one final 7–8 h day, and the Dynaport sensor was worn continuously for an additional 4 days to assess habitual free-living activity at 3 months post-SBC.

#### 2.3.1. Clinical Assessments

A standardized battery of validated clinical outcome measures, derived from the core measures recommended by the American Physical Therapy Association’s Academy of Neurological Physical Therapy (APTA, ANPT) [37,38], was administered at each of the five sessions. Motor and sensory functions of the affected limb were quantified using the motor domains of the Fugl-Meyer Assessment (FMA), which evaluates upper-extremity and lower-extremity impairments and synergistic movement patterns on a hierarchically ordered scale [39,40,41,42]. Manual dexterity and upper-extremity functional use were assessed with the Box and Blocks Test (BBT), which is a timed unilateral block-transfer task [43]. Self-reported functional recovery and stroke-related disability across eight domains, including strength, memory, emotion, communication, activities of daily living, mobility, hand function, and participation, were captured with the Stroke Impact Scale (SIS) [44]. Dynamic balance and gait function under challenging conditions were evaluated with the Functional Gait Assessment (FGA) [45], which is a 10-item performance scale incorporating balance perturbation tasks. Ambulatory speed was measured with the 10-Meter Walk Test (10 MWT) [46] at both comfortable and fast self-selected speeds, while functional walking endurance was assessed with the 6-Minute Walk Test (6 MWT) [47]. Static and dynamic postural stability were evaluated using the Berg Balance Scale (BBS) [48]. Bilateral grip strength was quantified using standardized grip dynamometry (GD) [49]. Finally, individualized occupational performance goals and perceived satisfaction were evaluated with the Canadian Occupational Performance Measure (COPM). All assessments were administered by a trained clinician following standardized administration protocols.

#### 2.3.2. Sensor-Based Multi-Day Monitoring

Continuous multi-day free-living movement was monitored using the McRoberts DynaPort MoveMonitor (McRoberts BV, The Hague, The Netherlands), a lower-back-mounted IMU containing a tri-axial accelerometer (range ±6 g, resolution ±1 mg) and tri-axial gyroscope (range ±100 °/s, resolution ±0.0069 °/s), sampled at 100 Hz. Raw data were stored in the manufacturer’s proprietary binary OMX format on the device’s internal SD card. The sensor was positioned at the lumbar region, with the y-axis oriented vertically downward in upright stance, such that the static gravitational vector was a = [ax, ay, az] = g [0, −1, 0]. This placement approximates whole-body center-of-mass motion and has demonstrated validity for the detection of gait bouts and the extraction of spatiotemporal gait parameters and nonlinear locomotor complexity metrics in real-world environments [20,21]. Although both accelerometer and gyroscope data were acquired, all signal-processing algorithms described below are based exclusively on accelerometer signals.

### 2.4. Signal-Processing Pipeline for Wearable Multi-Day Platform

Raw Dynaport sensor OMX files were processed using a multi-stage Python-(version 3.14.8) based signal analysis pipeline that was developed and validated for naturalistic multi-day lower-back accelerometry data. The pipeline is organized into six functional stages: (1) data ingestion and format conversion, (2) gait sequence detection, (3) initial contact and final contact event detection, (4) linear spatiotemporal gait parameter extraction, (5) nonlinear gait complexity analysis, and (6) structured output organization. Each stage is described below.

#### 2.4.1. Data Ingestion and Temporal Segmentation

**Figure 1:**
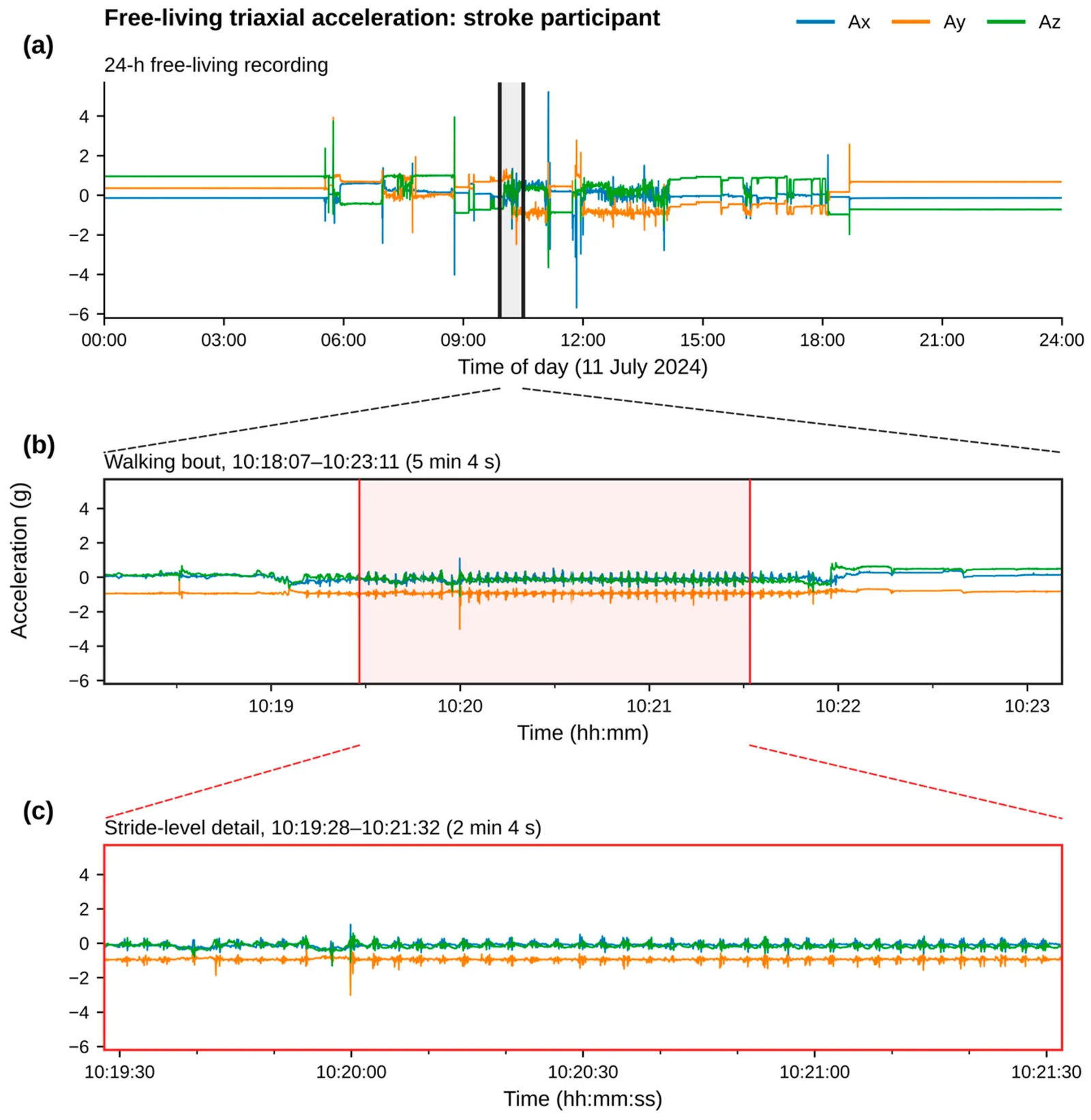
(**a**) Tri-axial acceleration data over 24 h; (**b**) zoomed-in portion showing walking pattern data; and (**c**) walking data identified for gait evaluation.

Binary OMX files were decoded via an Oct2Py-bridged Octave interface using the McRoberts vendor-supplied OMX_readFile library. Data were parsed in ten sequential packet chunks to manage memory overhead. The output accelerometer matrix contained four columns: MATLAB version 25.2 (R2025b) datenum timestamps (converted to Python datetime objects using the standard epoch offset) and tri-axial acceleration components (Ax, Ay, Az) in units of g. For longitudinal analysis, each continuous recording was automatically segmented into discrete 24 h epochs, enabling day-by-day characterization of ambulatory behavior across multi-day wear periods (Figure 1a–c).

#### 2.4.2. Gait Sequence Detection

**Figure 2:**
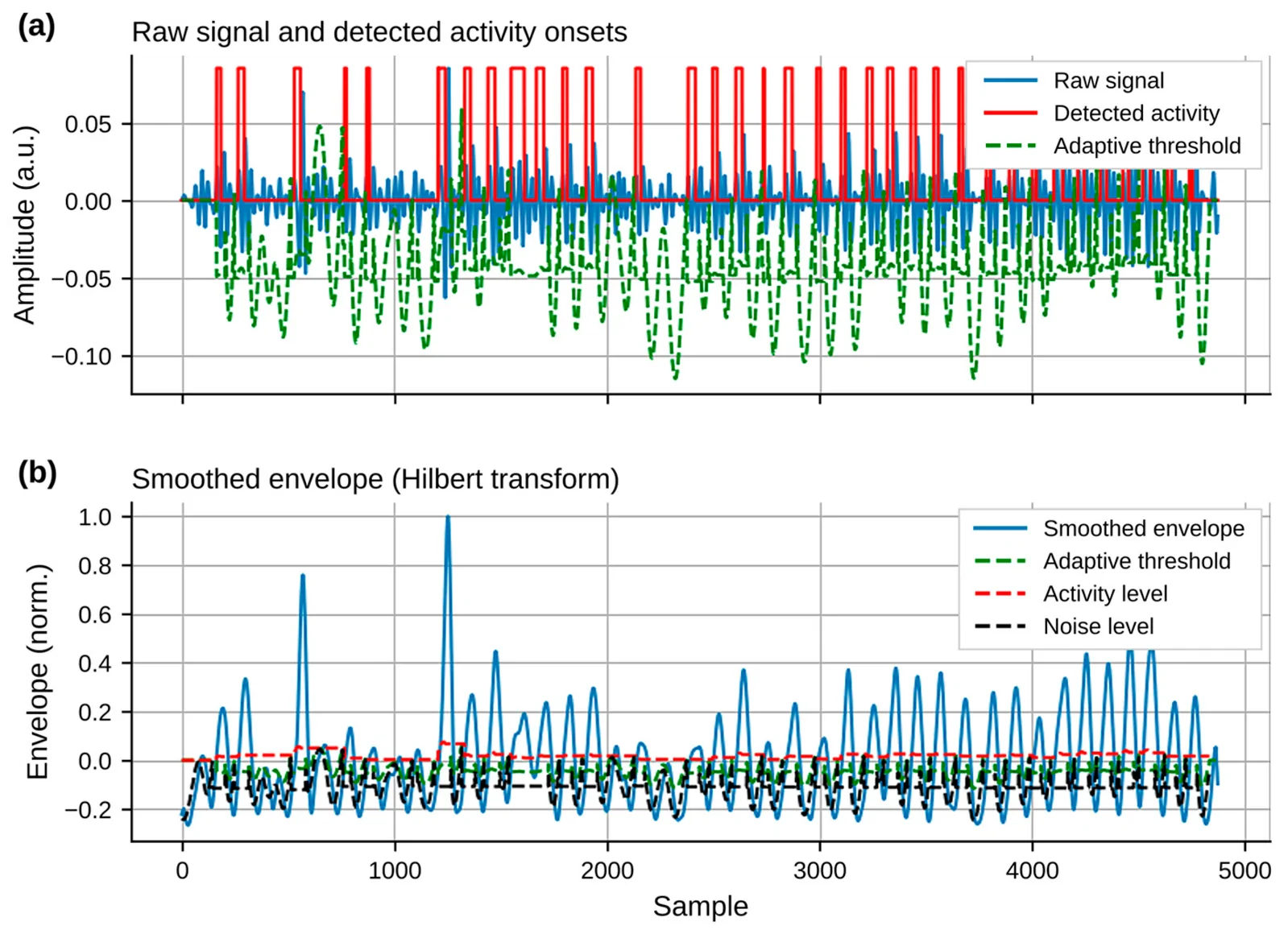
Walking epoch identification using sine-template convolution of band-pass-filtered (0.5–3.0 Hz) vertical and anterior–posterior acceleration components, with sliding-window variance thresholding (window = 300 samples, 50% overlap; activity threshold SD > 0.01) to classify walking epochs.

**Figure 3:**
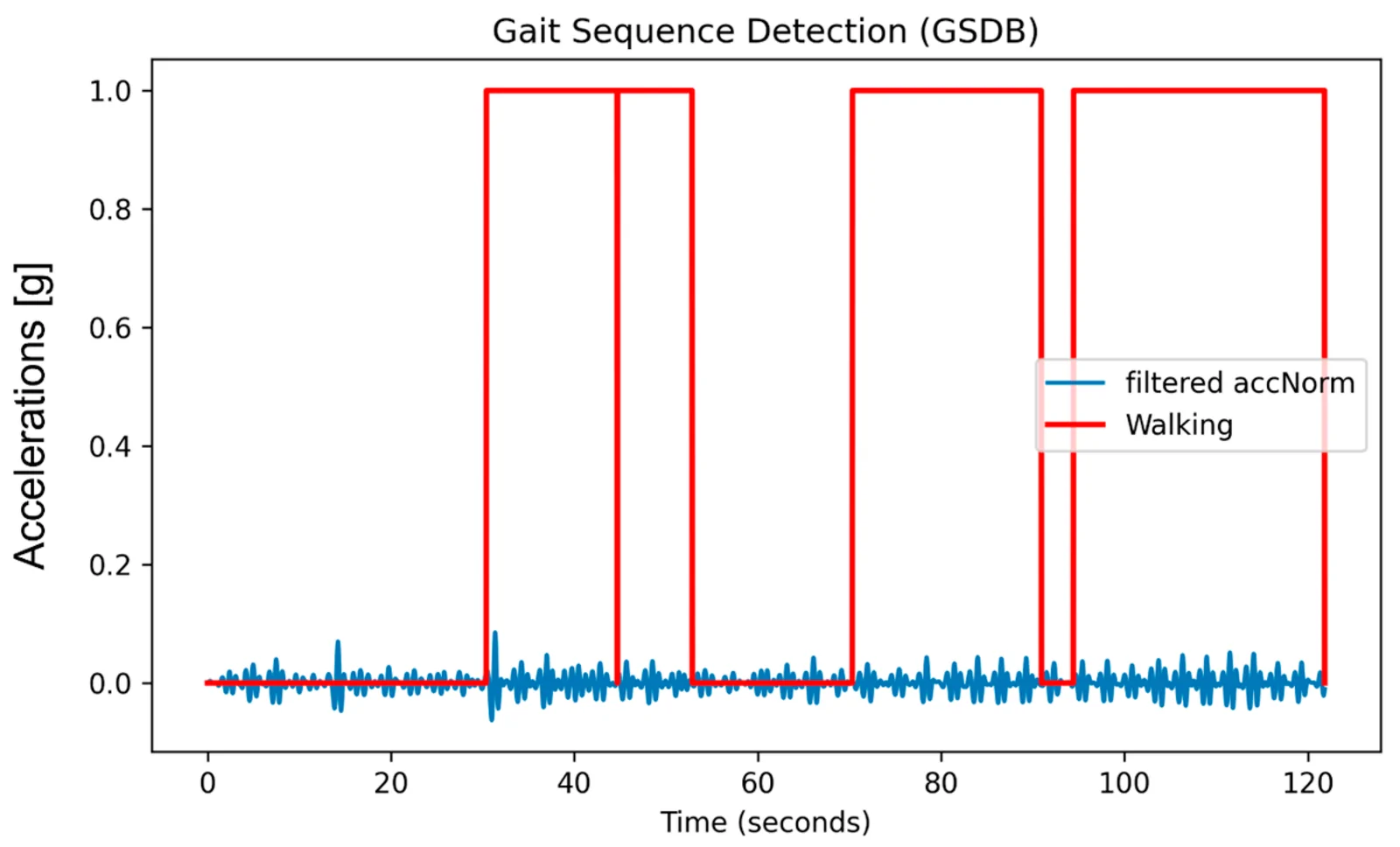
Gait sequence detection (GSD) using Mobilise-D algorithms for pathological gait. Unlike GSDA (sine-template convolution tuned to preserved gait), GSDB applies a multi-stage wavelet and Hilbert envelope pipeline that adaptively tracks the slower, lower amplitude, and more irregular step pulses characteristic of the pathological signal, illustrating the key processing difference between the two algorithms.

Two validated Mobilise-D gait sequence detection (GSD) algorithms were implemented to accommodate the heterogeneous gait profiles present in the stroke cohort. Algorithm GSDA, targeting relatively preserved gait, uses sine-template convolution of band-pass-filtered (0.5–3.0 Hz) vertical and anterior–posterior acceleration components, with sliding-window variance thresholding (window = 300 samples, 50% overlap; activity threshold SD > 0.01) to classify walking epochs [18,19] (Figure 2). Algorithm GSDB, optimized for slower and irregular hemiparetic gait patterns, operates at 40 Hz and applies nine sequential signal conditioning stages, including Savitzky–Golay smoothing, recursive IIR high-pass drift removal, double Gaussian second-derivative continuous wavelet transform (CWT), and iterative Gaussian kernel smoothing, followed by a Hilbert transform-adaptive envelope detector and step pulse train identification requiring a minimum of four concordant peaks per bout [18,20]. For each participant, the GSD algorithm was selected based on the severity of gait impairment; GSDB was applied to participants with moderate-to-severe hemiparetic gait, as assessed by clinical observation (Figure 3). To minimize reliance on qualitative judgment, algorithm selection was based on each participant’s baseline comfortable 10-Meter Walk Test (10 MWT) speed, a validated indicator of gait-impairment severity. GSDB was applied when the baseline speed fell below a comfortable-speed threshold of 0.6 m/s, consistent with Mobilise-D consortium’s guidance for distinguishing preserved from impaired real-world gait [18], and GSDA when it exceeded this cut-off. Accordingly, participant SA (0.21 m/s) was processed with GSDB, whereas the faster-ambulating participants SB and SC were processed with GSDA. This threshold was applied retrospectively; a prospectively pre-registered speed (or equivalent functional) cut-off should be adopted in larger trials. Output comprised a list of walking bout start and end timestamps, with step counts for each 24 h recording epoch.

#### 2.4.3. Initial Contact and Final Contact Detection

**Figure 4:**
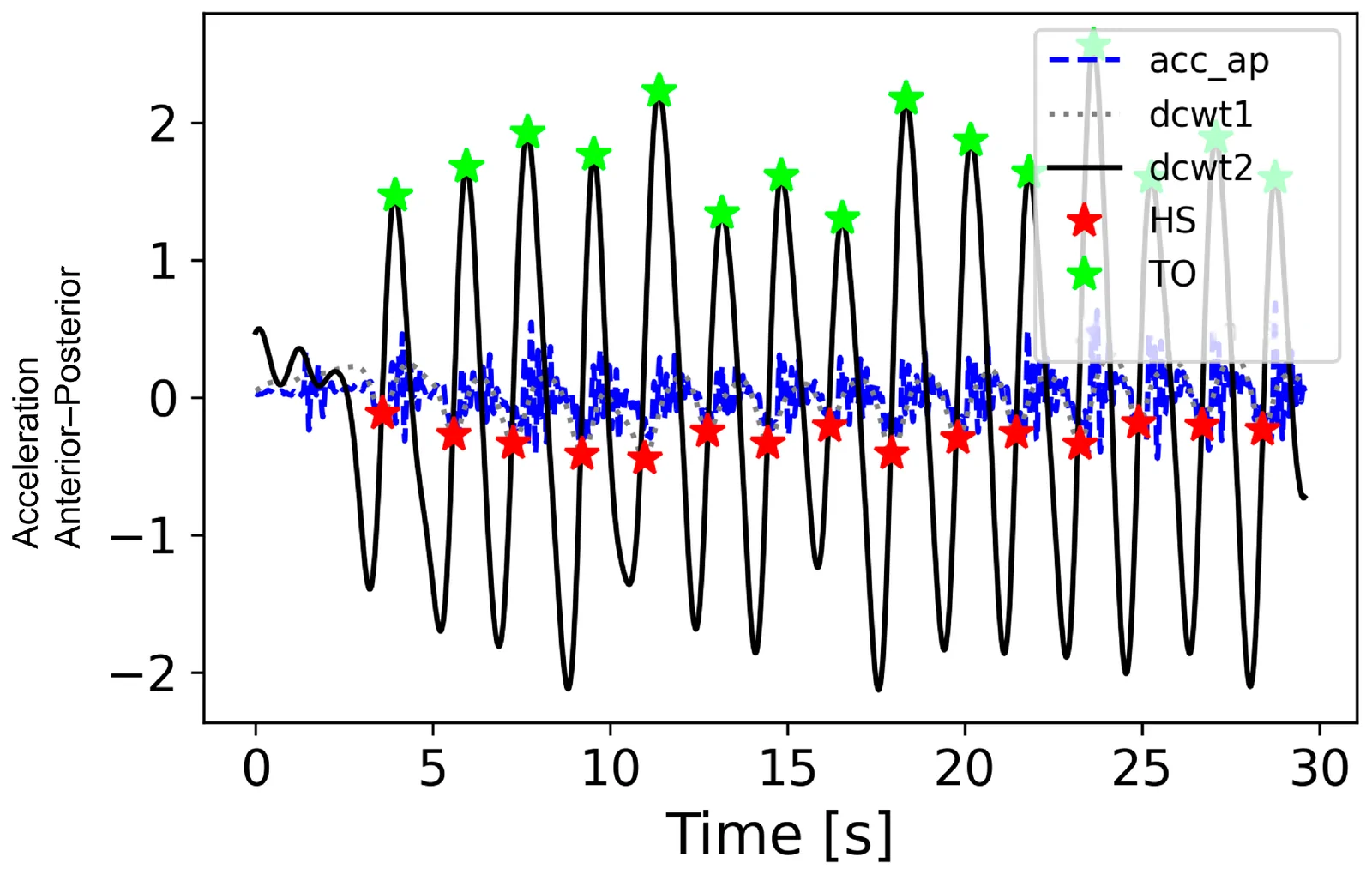
Heel strike (HS) and toe-off (TO) identification using wavelet analysis. Acceleration units are in ‘g’ and dcwt1 and dcwt2 units are in normalized amplitude units (a.u.).

Within each identified gait sequence, initial contact (HS; heel strike) and final contact (TO; toe-off) events were detected using the sd_algo_amc algorithm from the Mobilise-D Technical Validation Study algorithm library [18,21]. The vertical acceleration component was extracted, resampled to 40 Hz, and high-pass filtered with a recursive IIR filter to remove drift. A finite impulse response (FIR) low-pass filter (2–3 Hz, coefficients loaded from FIR-2-3Hz-40.mat) was applied after zero-padding to eliminate edge artifacts. The filtered signal was numerically integrated to approximate vertical velocity, and two successive Gaussian second-derivative CWT transformations (PyWavelets gaus2, scale = 9, fs = 40 Hz) were applied. Negative-region peaks of the first CWT output corresponded to HSs; positive-region peaks of the second CWT output corresponded to TOs. HS and TO timestamps were referenced to the original acquisition timeline for each gait bout (Figure 4).

#### 2.4.4. Linear Spatiotemporal Gait Parameters

Linear gait parameters were extracted from the anterior–posterior (Az) acceleration component at 100 Hz using a spectral cadence detection approach that identified walking bouts with step frequencies between 1.4 and 2.3 Hz. Within each walking bout, heel strikes and toe-offs were detected using Gaussian first-derivative wavelet-based detrending of the AP signal. Bilateral temporal parameters were derived from alternating foot contact sequences for both Foot 1 (less affected) and Foot 2 (more affected), including: gait cycle time (GCT), double-support time phases 1 and 2 (DST1, DST2), single-support time phases 1 and 2 (SST1, SST2), stance time (StT), and swing time (SwT). Step counts and walking bout durations were additionally extracted. Asymmetry between limbs was computed from paired bilateral parameters as the absolute difference normalized to the mean, providing a dimensionless index of inter-limb temporal symmetry across each session. To resolve any ambiguity between the two event detectors described above, all bilateral spatiotemporal parameters reported in Table 2 were derived exclusively from this 100 Hz anterior–posterior, Gaussian first-derivative wavelet method (Section 2.4.4); the Mobilise-D sd_algo_amc detector (Section 2.4.3) was used only to provide validated initial and final contact events for gait sequence verification and for timing the nonlinear analyses, and therefore did not contribute to Table 2. Phases were defined per stride relative to ipsilateral events: gait cycle time (GCT) is the interval between two consecutive ipsilateral heel strikes (100% of the cycle); swing time is the interval from ipsilateral toe-off to the next ipsilateral heel strike; stance time is the remainder (GCT minus swing); double-support time is the sum of the two both-feet-in-contact intervals; and single-support time is the interval when only the reference limb contacts the ground, which, by definition, coincides with contralateral swing. Because single-support and swing thus describe simultaneous events on opposite limbs, and because each phase is summarized as an independent per-limb median across bouts, the phase durations are not arithmetically additive to GCT in seconds; to make the partition interpretable, phase durations are additionally expressed as proportions of the gait cycle (%GCT) in Table 2.

#### 2.4.5. Nonlinear Gait Complexity Analysis

Nonlinear dynamical analysis was performed on the resultant acceleration signal (||a|| at 100 Hz) across each walking bout to characterize locomotor complexity, regularity, and local dynamic stability. Phase-space reconstruction parameters were first estimated: the time delay (τ) was determined from the first local minimum of the Average Mutual Information (AMI) function (maximum lag = 35 samples; Stergiou method), and the embedding dimension (m) was estimated via the False Nearest Neighbors (FNN) algorithm (MaxDim = 30, Rtol = 15, Atol = 2; [23]). Entropy-based regularity was quantified using approximate entropy (ApEn; m = 2, r = 0.2 SD), sample entropy (SampEn; m = 2, r = 0.2 SD), symbolic entropy (SymEn; binary alphabet, symbol length L = 2), and permutation entropy (PermEn; m = 2, delay = τ). Multi-timescale complexity was characterized via multi-scale entropy (MSE), composite MSE (CMSE), and refined composite MSE (RCMSE), computed across coarse-graining scales τ = 1–20 with m = 2 and r = 0.2 [24]. Scale 3 was selected because it provides a modest temporal coarse grain while preserving sufficient data points for entropy estimation from the IMU gait segments. This scale may reduce sensitivity to high-frequency sensor noise while retaining short-timescale fluctuations associated with gait dynamics. Local dynamic stability was assessed using the largest Lyapunov exponent estimated by both the Rosenstein algorithm (yielding short-term and long-term divergence rates) and the Wolf algorithm. The recurrence structure was quantified via recurrence quantification analysis (RQA), providing ten metrics, which include percent recurrence (%REC), percent determinism (%DET), mean and maximum diagonal line lengths (MeanL, MaxL), diagonal line entropy (EntrL), laminarity (%LAM), mean and maximum vertical line lengths (MeanV, MaxV), vertical line entropy (EntrV), and waveform entropy (EntrW). RQA was computed on the phase-space-reconstructed attractor using a fixed recurrence radius of r = 0.2 multiplied by the signal standard deviation. We note that this radius, adopted from healthy-gait conventions, proved too permissive for the highly periodic hemiparetic signals in this cohort, saturating %DET and %LAM at 100% (ceiling effect); a smaller, cohort-calibrated radius (or a fixed recurrence-rate criterion) should be adopted in future work to restore sensitivity of these metrics. Together, these 24 nonlinear metrics characterize the multidimensional structure of locomotor dynamics in ways that complement conventional temporal gait parameters.

## 3. Results

### 3.1. Longitudinal Aggregation and Outcome Extraction

Walking bouts longer than 30 s were identified from each 24 h recording epoch for analysis. Day-level gait metrics were summarized across valid bouts using median values, and session-level summaries were aligned across four visits: pre-SBC baseline (V1), SBC Week 1 (V2), SBC Week 2 (V3), and 1-month follow-up (V4). The nonlinear IMU analyses reported here span the continuous DynaPort monitoring block that ran uninterrupted from the pre-SBC baseline through to the 1-month follow-up (V1–V4); this window was selected because it provides temporally contiguous, intervention-adjacent recordings suitable for within-subject trajectory analysis. Session 5’s DynaPort recording, obtained at 3 months post-SBC, was collected but is not included in the present nonlinear analysis, as this falls outside this continuous monitoring block; the 3-month time point is instead represented in the clinical and patient-reported outcomes (Tables 5–7). The case series included three community-dwelling stroke survivors (SA, SB, and SC) who were assessed with lower-back-mounted Dynaport IMUs. Given the small sample size (n = 3), the results are presented descriptively as individual trajectories and percent changes from baseline.

### 3.2. Spatiotemporal Gait Parameter

Spatiotemporal gait outcomes (GC duration, DST, SST, and swing time; Table 2) showed substantial inter-individual variability at baseline and across visits.

Participant SA presented with markedly prolonged baseline GC (5.72 s, bilateral average), consistent with moderate-to-severe post-stroke gait impairment. GC decreased by 38.2% at V2, partially recovered at V3 (−10.2%), and remained reduced at V4 (−33.2%), suggesting a durable increase in walking speed. DST decreased by 44.1% at V2, indicative of reduced reliance on bilateral support, but had increased substantially by V4 (+80.4%), possibly related to gradual decline when away from SBC, which warrants further investigation. SST and swing time followed similar reductions during SBC (V2: ~−37 to 39%) without full retention at follow-up. Total steps increased modestly during SBC (+12.5% at V2–V3) and returned to near baseline by V4. Here, total steps denotes the median number of steps per instrumented 24 h day (aggregated across all valid walking bouts) and not steps within the 30 s analyzed bouts. The disproportionately large reduction in SA’s swing time at V4 (−68.0%), relative to the modest change in cycle duration (−10.2%), should be interpreted with caution, as wavelet-based event detection is less reliable for very slow, highly asymmetric hemiparetic gait, and may constitute a detection artifact.

Participant SB’s baseline GC (1.03 s) was near the normative range, reflecting a faster pre-intervention walking pace. GC had increased substantially by V2 (+135.4%), accompanied by large increases in DST (+193.0%) and SST (+135.4%), suggesting the adoption of a deliberate, slow-controlled gait pattern during early SBC, possibly in response to retraining cues or supervised attentional focus emphasizing the quality of movement over velocity. All parameters largely returned toward baseline by V3 and V4.

Participant SC demonstrated mildly elevated baseline GC (1.29 s). Changes across visits were of a smaller magnitude than in SA or SB. GC increased modestly by V2 (+8.6%) and V3 (+14.9%), returning near baseline by V4 (−2.9%). DST remained relatively stable (range: −4.4 to +6.5%). SST and swing time showed incremental increases during SBC (SST V3: +13.4%; swing time V3: +17.7%), suggesting modest improvements in single-limb stability that did not fully persist at follow-up.

### 3.3. Signal Entropy and Gait Complexity

Entropy measures (ApEn, SampEn, symbolic entropy, permutation entropy) quantify gait signal regularity; higher values reflect greater complexity, a hallmark of healthy locomotion (Table 3).

#### 3.3.1. Approximate and Sample Entropy

SA showed progressive complexity increases during SBC, peaking at V3 (ApEn +39.6%; SampEn +56.4%) with partial retention by V4 (ApEn +19.7%; SampEn +36.9%). SB demonstrated the inverse pattern ApEn declined during SBC (V3: −20.3%) before returning to near baseline by V4 (+5.6%), consistent with the deliberate, controlled gait strategy observed in the spatiotemporal data. SC showed transient increases at V2 (ApEn +24.2%; SampEn +21.7%) that were attenuated by V4 (Table 3).

#### 3.3.2. Symbolic and Permutation Entropy

Symbolic entropy showed greater between-participant variability, with no consistent directional trend. Permutation entropy remained near ceiling (≥0.985) across all participants and visits (changes < 1%), indicating insufficient sensitivity to detect intervention-related changes in this sample and was excluded from further interpretation.

#### 3.3.3. Lyapunov Exponents

Long-term LyEs (larger positive values correspond to greater gait instability) showed divergent individual patterns: SA exhibited near-zero values at V2 (0.002; −95.8%), suggesting transiently stabilized dynamics during early SBC, while SC showed a progressive reduction through to V3 (−48.6%), with a partial return by V4 (−23.2%), indicating more sustained instability reduction. The most consistent cross-participant finding was a substantial increase in Wolf LyEs during SBC Week 2 (V3)—SA +54.1%, SB +62.4%, and SC +52.2%—as the only convergent nonlinear signal across all three participants, potentially reflecting heightened gait challenge and motor adaptation during the latter phase of the program (Table 3).

#### 3.3.4. Recurrence Quantification Analysis

%DET and %LAM were uniformly 100% across all participants and visits (ceiling effect) and were not interpreted further. Because this saturation limited the ability of %DET and %LAM to detect longitudinal changes, both metrics were explicitly excluded from interpretation rather than simply omitted from the tables. %REC decreased during SBC in SA (V3: −29.7%) and SB (V3: −39.2%), suggesting a reduced gait stereotype during the intervention, with a partial return toward baseline by V4. SC showed minimal %REC variation across visits (range: −16.4 to +10.0%). The diagonal line length (MeanL) remained stable across all participants (changes within ±5%), indicating consistent periodic structure in the gait attractor (Table 4).

### 3.4. Multi-Scale Entropy

RCMSE was computed at scale 3, reflecting medium-timescale trunk acceleration complexity; values near 1.0 are characteristic of healthy locomotion. SA showed a modest early increase at V2 (+5.9%) followed by reductions by V3 and V4 (−16.4% and −18.1%, respectively), indicating a net complexity decline at follow-up. SB demonstrated a progressive increase from V3 onward, reaching +5.5% by V4, suggesting modest but durable post-SBC gains. SC exhibited the largest trajectory in this dataset: RCMSE increased 39.3% by V2 and 47.2% by V3 before partially returning toward baseline by V4 (−7.2%). CMSE and MSE showed identical patterns, confirming the robustness of the scale-3 findings.

### 3.5. Clinical Outcomes, Patient-Reported Performance, and Limb Symmetry

Clinical and patient-reported outcomes are summarized in Table 5, Table 6 and Table 7; APDM-derived limb symmetry ratios are presented in Table 8. Across upper-extremity functions, SC demonstrated the clearest gains, with UEFM improving from 43 to 54 after one month and paretic grip strength increasing from 12.1 to 14.5 kg, although both showed partial regression after three months (UEFM 52; grip 11.1 kg) (Table 5). SA and SB showed limited paretic hand changes, with BBT paretic remaining at zero for SA throughout. For mobility and balance, despite their severely impaired baseline gait (10 MWT: 0.21 m/s), SA showed notable improvements in balance (BERG: 24 to 32) and functional gait (FGA: 0 to 10) after three months, suggesting gains in gait quality that were not fully captured by walking speed (Table 6). SC improved on the 10 MWT (1.44 to 1.68 m/s) and 6 MWT (329 to 445 m) after one month, with a partial return after three months. COPM scores reflected clinically meaningful changes (≥2 points) for most goals at the one month mark, particularly for SB (walk faster, grasp/release: performance +5 points) and SC (computer mouse, utensil use), with partial regression after three months (Table 7). APDM wrist angular velocity asymmetry was persistently reduced in SA (A/UA range: 0.19–0.45), consistent with severe paretic upper-limb impairment. Foot acceleration ratios were near-symmetric across all participants (≥0.98), while foot angular velocity asymmetry showed greater variability; SA’s foot gyro A/UA improved from 0.62 to a peak of 1.31 at V3 before returning to 0.76 by V4, and SB demonstrated stable, moderate asymmetry across all visits (0.76–0.84) (Table 8).

## 4. Discussion

A foundational methodological contribution of this study is the demonstration that a single lumbar-mounted IMU device, one that weighs less than 20 g and can be worn and deployed in any ambulatory environment, simultaneously generated five analytically distinct gait characterizations (spatiotemporal, entropy, Lyapunov, RQA, MSE) that collectively detected intervention effects not visible in any individual metric alone. This finding directly addresses one of the most persistent translational gaps in stroke rehabilitation science, since there is paucity of validated, clinically accessible tools capable of capturing the full complexity of gait recovery outside the laboratory [12,50]. Conventional laboratory-based gait analysis, while mechanistically informative, is logistically prohibitive for repeated assessments across a rehabilitation episode and entirely inaccessible for in-community or in-home monitoring. Wearable IMU technology resolves this access barrier, but its clinical utility has been substantially constrained by analytical pipelines that report only spatiotemporal parameters, such as locomotor speed and cadence, while discarding the nonlinear structure of the acceleration signal that encodes coordinative organization, neuromuscular adaptability, and postural regulation [51,52].

The present study operationalizes an expanded analytical architecture layered onto a single lumbar IMU signal that is, in principle, fully deployable in a home or community setting. The Dynaport system’s validated algorithms for extracting gait cycle duration, double-support time, single-support time, and swing time from the lumbar acceleration signal provide the standardized spatiotemporal foundation upon which the nonlinear analyses of SampEn, Wolf LyE, Long LyE, RQA, and MSE are layered without requiring additional instrumentation. This pipeline architecture carries direct implications for future home-based monitoring protocols since the signal acquired passively during a 2 min household walk could, in principle, yield the same five-domain output reported here. A clinical monitoring protocol that reports only spatiotemporal parameters, as is common in current clinical IMU applications, would have missed the convergent Wolf LyE signal (the most consistent cross-participant finding), participant SA’s entropy gains, and participant SC’s multi-scale complexity increase, which may be the subclinical gait changes that are most likely to precede or predict later functional improvements [29,53].

The lower-back placement is specifically well-suited for this multi-domain analytical framework, because the lumbar acceleration signal integrates contributions from bilateral lower-extremity kinematics, trunk sway, and whole-body center-of-mass dynamics, providing a global view of locomotor control that single-limb sensors cannot replicate [51,54]. This integrative property is particularly relevant in hemiparetic stroke gait, where asymmetric lower-limb mechanics propagate upward through the kinetic chain and manifest as altered trunk oscillation patterns detectable in the lumbar acceleration signal [55]. The bilateral asymmetry implicit in stroke gait is visible in the Dynaport Foot 1 vs. Foot 2 data and in the APDM affected-to-unaffected ratios, confirming that the lumbar IMU integrates these asymmetric contributions into a single composite signal whose nonlinear structure reflects the emergent coordinative behavior of the full locomotor system.

In this study with descriptive case series, we examined gait dynamics in three chronic stroke survivors (SA, SB, and SC) across four time points spanning a two-week Stroke Boot Camp (SBC) intervention with a one-month follow-up. The three participants represented a wide spectrum of post-stroke gait impairment at baseline. This distribution has fundamentally shaped the interpretation of all subsequent findings and directly motivates the case for individualized, continuous in-home monitoring rather than periodic clinic-based assessment. SA’s bilateral-average gait cycle duration of 5.72 s places this participant well outside the normative range for community-dwelling older adults (approximately 0.8 s or above) and is consistent with severe hemiparetic gait requiring substantial compensatory movement strategies [56]. SB and SC, with baseline gait cycle durations of 1.03 s and 1.29 s, respectively, represent mild-to-moderate impairment levels that are more characteristic of limited community ambulation. This pronounced inter-participant heterogeneity is consistent with the well-recognized variability of chronic stroke presentation and underscores that rehabilitation-related gait changes are highly individualized, even within a common intensive intervention framework [57,58]. Pooling these participants into group-level statistics would produce averages that represent no individual’s actual gait trajectory and would obscure mechanistically distinct adaptation patterns. Individual trajectory analysis, supported by percent changes from baseline, is therefore not a concession due to a small sample size; it is the methodologically appropriate strategy for characterizing heterogeneous stroke recovery [59,60]. Importantly, the case for individualized monitoring has a clear practical implication: home-based IMU deployment enables dense longitudinal sampling of gait across the full recovery trajectory, allowing daily or weekly assessment of change that is not feasible within clinic-based protocols alone [57]. Our data strongly suggest that future IMU-based stroke rehabilitation trials should pre-specify baseline gait speed, or an equivalent functional classification, as a primary stratification variable instead of analyzing stroke survivors as a homogeneous group.

Among all examined metrics, Wolf’s Lyapunov exponent (LyE) was the most consistent cross-participant finding, increasing in all three participants during SBC Week 2 (SA: +54.1%, SB: +62.4%, SC: +52.2%). This pattern of convergence is striking given the pronounced baseline heterogeneity in gait and the fact that no other nonlinear metric demonstrated comparable consistency. Wolf LyE estimates the maximal Lyapunov exponent from the reconstructed gait attractor and reflects how rapidly nearby trajectories diverge in phase space [61]. In gait, higher positive LyE values may reflect either pathological instability or, within a structured rehabilitation context, increased motor exploration consistent with active reorganization of coordination.

Several mechanistic interpretations are plausible. First, SBC Week 2 likely imposed a greater task intensity and complexity than Week 1, which could expand gait dynamics and increase local divergence [62]. Second, the increase may reflect adaptive motor exploration during skill reacquisition, consistent with the principle that transient increases in movement variability support the search for more effective coordination strategies during motor learning [29,63]. The divergent follow-up patterns, with SB (+17.1%) and SC (+5.9%) approaching baseline and SA (+52.9%) remaining elevated, suggest that the most severely impaired participant may require a longer consolidation period before gait dynamics stabilize around a new attractor state. Accordingly, if Wolf LyE remains elevated after intensive rehabilitation in more severely impaired individuals, home-based IMU monitoring may serve as an objective indicator of continued motor consolidation or, alternatively, of instability that warrants clinical reassessment [64,65]. Future work should prospectively examine the relationship between Wolf LyE and session-level task complexity to determine whether it can serve as a real-time marker of training challenge and participant engagement, with potential for future streaming from a home-worn IMU to a supervising clinician.

The long-term Lyapunov exponent, reflecting stride-to-stride orbital stability, showed more individualized responses. SA demonstrated a near-zero Long LyE at V2 (0.002; −95.8%), consistent with highly periodic, stereotyped gait early in training. SC showed progressive reductions by V2 and V3 (−34.2% and −48.6%), with only partial return at follow-up (−23.2%), suggesting sustained improvement in stride-to-stride stability. The combination of increased Wolf LyE and reduced Long LyE at V3 in SC suggests a mechanistically meaningful dissociation in which gait became more locally exploratory within individual strides while becoming more orbitally stable across strides—a pattern that has previously been observed during healthy adaptation to novel perturbations [64] and is interpretable as a signature of adaptive motor reorganization. Capturing this dissociation requires exactly the multi-domain analytical pipeline described in this study.

At scale 3, multi-scale entropy (MSE) provided one of the most physiologically distinctive findings in the dataset. SC showed large increases in RCMSE by V2 (+39.3%) and V3 (+47.2%), which are the largest entropy-domain changes observed across participants, metrics, and visits. The near-identical pattern across RCMSE, CMSE, and MSE reduces the likelihood of computational artifact given their differing approaches to boundary conditions and template matching [66,67]. Since scale 3 in MSE reflects medium-timescale fluctuations linked to inter-stride postural regulation, anticipatory balance control, and whole-body coordination [68], these findings suggest that SBC substantially increased regulatory complexity in a physiologically meaningful temporal band. The near-return to baseline at follow-up (−7.2%) is consistent with partial loss of training-induced complexity after withdrawal of intensive supervised practice [69], and raises a clinically important question that in-home monitoring is well-positioned to address: can continued home-based practice or structured community walking in the weeks after intensive intervention sustain entropy gains, or is booster SBC rehabilitation necessary? In contrast, SB demonstrated a modest but progressive MSE increase through follow-up, suggesting the most durable retention profile and potentially reflecting a ceiling effect in higher-functioning participants. SA showed early gains in entropy, but this was followed by declines by V3 and V4 despite the preservation of single-scale entropy gains, suggesting a dissociation between local within-stride variability and regulatory complexity at intermediate timescales that warrants further longitudinal investigation. Together, these divergent MSE trajectories illustrate that post-rehabilitation gait complexity is highly individualized and is not adequately captured by any single metric or assessment timepoint, thus precisely the rationale for continuous, home-based multi-domain monitoring rather than discrete clinic visits.

Clinical and patient-reported outcomes supported the lumbar IMU findings across multiple domains. SA’s severe baseline impairment was consistent with a prolonged gait cycle duration and elevated %REC, indicating that nonlinear metrics tracked true gait severity. SA’s improvement in FGA (0 to 10 at three months), combined with retained SampEn gains (+36.9% at V4), raises the possibility that greater gait complexity may relate to improved functional gait quality; this hypothesis is testable by using longitudinal in-home monitoring designs that pair passive IMU data collection with periodic clinical assessments [70]. SC showed the strongest multi-domain convergence, with gains in RCMSE (+47.2% by V3), 10 MWT (+16.7% after one month), and COPM performance, suggesting that nonlinear IMU metrics and functional outcome measures may be tracking the same underlying recovery process from complementary perspectives. SB’s stable clinical status and modest IMU changes suggest a possible ceiling effect in higher-functioning participants, which is an important design consideration for future in-home monitoring trials that should pre-stratify by baseline function to ensure sensitivity across the full impairment spectrum. APDM symmetry measures further supported severity differences, particularly SA’s persistently low wrist angular velocity affected-to-unaffected ratio and the coincidence of peak foot gyro asymmetry with the Week 2 Wolf LyE increase, suggesting concurrent reorganization of lower-limb symmetry and global gait dynamics detectable from a single lumbar sensor.

## 5. Limitations

Several limitations must be acknowledged. First, with only three participants, findings are descriptive and hypothesis-generating rather than inferential; replication in larger stratified cohorts is required before any metric is advanced as a clinical endpoint. Second, the absence of a control group precludes definitive attribution of changes to SBC, although temporal convergence across participants and metrics argues against chance. Third, a single lumbar IMU cannot separate affected from unaffected limb contributions, and Foot 1/Foot 2 assignments are inferred rather than directly measured, introducing asymmetry estimation uncertainty. Fourth, within-visit (on the same day) gait variability was not analyzed despite its clinical relevance for in-home monitoring contexts where visit-to-visit signal stability directly affects interpretability [71]. Because each session included 3–4 consecutive days of recording, we calculated the median across days to reduce day-to-day fluctuations. However, we did not formally separate variability between days within a session from variability across sessions. The main longitudinal change particularly the 52–62% increase in Wolf’s Lyapunov exponent at Week 2 was considerably larger than the day-to-day variability typically reported for lumbar IMU gait measures. This suggests that the observed change may reflect the intervention rather than the noise measurement. Nevertheless, future studies should formally compare within-session and between-session variability to confirm the sensitivity of these measures to longitudinal change. Fifth, important clinical covariates, such as stroke subtype, lesion location, chronicity, and medication status, were not systematically incorporated, limiting mechanistic interpretation. Finally, ceiling effects in %DET and %LAM indicate that RQA parameters were not optimized for this population and should be refined prior to use as primary outcomes in future trials.

## 6. Future Directions

Our findings support several high-priority directions for future investigation. Larger, prospectively stratified cohorts are needed to formally test the complexity-change hypotheses generated here and to permit repeated-measures analyses with adequate power for nonlinear gait metrics as primary endpoints. In future, we would like to validate the analytical pipeline demonstrated here with five-domain nonlinear characterization from a single lumbar IMU in a home-based monitoring context, where participants wear the device during unstructured daily ambulation across a multi-week rehabilitation episode [72]. This would directly test whether the intervention-sensitive metrics identified here (Wolf LyE, SampEn, RCMSE) retain their sensitivity under the lower signal control and greater environmental variability inherent to free-living measurement. Longer follow-ups at three and six months would clarify retention of entropy and Lyapunov-based changes and determine whether within-intervention complexity gains predict long-term functional outcomes. The consistent Week 2 Wolf LyE increase also warrants prospective validation as a marker of training engagement by correlating it with session-level task complexity and can be further communicated to supervising clinicians as an objective engagement index.

## 7. Conclusions

This proof-of-concept case series of three stroke survivors shows that a single lumbar-mounted Dynaport IMU can capture multi-domain gait dynamics during intensive stroke rehabilitation and identify changes not evident from conventional spatiotemporal metrics alone. The participants demonstrated heterogeneous baseline gait patterns and divergent treatment responses, underscoring the importance of individual-level analysis in small stroke rehabilitation samples. The most consistent finding was a 52–62% increase in Wolf’s Lyapunov exponent during SBC Week 2 across all participants, suggesting a potential IMU-based marker of gait attractor perturbation during intensive training. Participant-specific entropy changes, including SC’s increase in scale-3 multi-scale entropy and SA’s sustained SampEn gain at follow-up, further suggest that nonlinear lumbar IMU metrics may detect gait reorganization across multiple temporal scales. Together with the accompanying clinical improvements, these results provide a rationale and analytic foundation for larger controlled studies evaluating in-home IMU-derived nonlinear gait complexity as a rehabilitation outcome.

## Figures and Tables

**Table 1:** Longitudinal Data Collection Schedule for the Stroke Boot Camp Sensor Study.

Session	Time Point	APDM (Wrist/Ankle)	Dynaport (Lower Back)	Clinical Assessments
S1	1 week pre-SBC	1 day (7–8 h)	4 days continuous	FMA, BBS, FGA, 10 MWT, 6 MWT, BBT, SIS, COPM, GD
S2	Day 1 of SBC	1 day (7–8 h)	Continuous (worn throughout SBC)	FMA, BBS, FGA, 10 MWT, 6 MWT, BBT, SIS, COPM, GD
S3	Last day of SBC (~2 weeks after S2)	1 day (7–8 h)	Continuous (1 month post-SBC; mid-point recharge at 14 days)	FMA, BBS, FGA, 10 MWT, 6 MWT, BBT, SIS, COPM, GD
S4	1 month post-SBC	1 day (7–8 h)	4 days continuous	FMA, BBS, FGA, 10 MWT, 6 MWT, BBT, SIS, COPM, GD
S5	3 months post-SBC	1 day (7–8 h)	4 days continuous	FMA, BBS, FGA, 10 MWT, 6 MWT, BBT, SIS, COPM, GD

Note. APDM = wrist- and ankle-mounted inertial sensors (bilateral, 4-sensor configuration); Dynaport = lower-back-mounted DynaPort accelerometer/gyroscope; Continuous = uninterrupted wear across waking hours; day = standardized 7–8 h instrumented day in a natural environment; SBC = Stroke Boot Camp; FMA = Fugl-Meyer Assessment; BBS = Berg Balance Scale; FGA = Functional Gait Assessment; 10 MWT = 10-Meter Walk Test; 6 MWT = 6-Minute Walk Test; BBT = Box and Blocks Test; SIS = Stroke Impact Scale; COPM = Canadian Occupational Performance Measure; GD = grip dynamometry.

**Table 2:** Per-limb spatiotemporal gait parameters (Foot 1/Foot 2), expressed as means across walking bouts.

Participant	Parameter (Foot 1/Foot 2)	V1 Baseline	V2 SBC Wk1	V3 SBC Wk2	V4 1-Mo F/U
SA	Gait cycle, s	5.68/5.76	3.55/3.52	5.15/5.13	3.85/3.79
	Stance, %GCT	61/62	62/59	62/63	89/74
	Swing, %GCT	39/38	38/41	38/37	11/26
	Double support, %GCT	22/25	22/21	25/25	59/67
	Single support, %GCT	39/37	41/38	38/37	30/7
SB	Gait cycle, s	1.03/1.03	2.40/2.43	1.11/1.11	0.85/0.84
	Stance, %GCT	70/54	66/64	63/67	70/60
	Swing, %GCT	30/46	34/36	37/33	30/40
	Double support, %GCT	22/25	34/24	25/35	33/28
	Single support, %GCT	47/29	31/39	38/32	37/33
SC	Gait cycle, s	1.29/1.28	1.40/1.39	1.48/1.48	1.25/1.25
	Stance, %GCT	63/63	63/61	59/64	62/63
	Swing, %GCT	37/37	37/39	41/36	38/37
	Double support, %GCT	26/25	24/25	26/22	25/25
	Single support, %GCT	36/38	39/36	34/42	37/38

Note. Each cell shows Foot 1/Foot 2. Values are means across all detected walking bouts at each visit (free-living, self-selected speeds); percentages are relative to that limb’s gait cycle. Because a single lumbar IMU cannot establish limb identity, Foot 1 and Foot 2 are reported as unlabeled sides rather than paretic vs. non-paretic; for the same reason, and because free-living walking occurs at variable speeds, parameters are reported per limb rather than as a bilateral average. Values for V2 to V4 are followed by the percent change from baseline in parentheses. V1 = baseline (pre-SBC); V2 = SBC Week 1; V3 = SBC Week 2; V4 = 1-month follow-up. SBC = Stroke Boot Camp; s = seconds; F/U = follow-up.

**Table 3:** Signal entropy and nonlinear gait dynamics across four assessment visits.

	V1 Baseline	V2 SBC Week 1	V3 SBC Week 2	V4 1-Month F/U
**Participant SA**				
ApEn	0.360	0.473 (+31.4%)	0.503 (+39.7%)	0.431 (+19.7%)
SampEn	0.321	0.416 (+29.6%)	0.502 (+56.4%)	0.439 (+36.8%)
Long-Term LyE	0.048	0.002 (−95.8%)	0.096 (+100.0%)	0.019 (−60.4%)
Wolf LyE	21.72	26.08 (+20.1%)	33.48 (+54.1%)	33.21 (+52.9%)
**Participant SB**				
ApEn	0.505	0.427 (−15.4%)	0.403 (−20.2%)	0.534 (+5.7%)
SampEn	0.574	0.490 (−14.6%)	0.566 (−1.4%)	0.576 (+0.3%)
Long-Term LyE	0.234	0.194 (−17.1%)	0.307 (+31.2%)	0.132 (−43.6%)
Wolf LyE	20.33	23.91 (+17.6%)	33.01 (+62.4%)	23.81 (+17.1%)
**Participant SC**				
ApEn	0.314	0.390 (+24.2%)	0.355 (+13.1%)	0.303 (−3.5%)
SampEn	0.355	0.432 (+21.7%)	0.341 (−3.9%)	0.354 (−0.3%)
Long-Term LyE	0.284	0.187 (−34.2%)	0.146 (−48.6%)	0.218 (−23.2%)
Wolf LyE	18.74	33.37 (+78.1%)	28.52 (+52.2%)	19.85 (+5.9%)

Note. Values represent session means. Values for V2–V4 are followed by percent changes from baseline in parentheses. ApEn = approximate entropy; SampEn = sample entropy; Long-Term LyE = long-term Lyapunov exponent; Wolf LyE = Wolf’s Lyapunov exponent. Higher entropy values indicate a greater gait signal complexity. Permutation entropy (range: 0.985–1.000 across all visits) and Short-term LyE are not shown due to negligible variability.

**Table 4:** Recurrence quantification analysis and multi-scale entropy across four assessment visits.

	V1 Baseline	V2 SBC Week 1	V3 SBC Week 2	V4 1-Month F/U
**Participant SA**				
%REC	21.87	18.86 (−13.8%)	15.38 (−29.7%)	17.46 (−20.2%)
MeanL	1456.8	1465.8 (+0.6%)	1465.3 (+0.6%)	1519.8 (+4.3%)
RCMSE	0.919	0.973 (+5.9%)	0.768 (−16.4%)	0.753 (−18.1%)
**Participant SB**				
%REC	6.07	4.94 (−18.6%)	3.69 (−39.2%)	6.86 (+13.0%)
MeanL	1496.5	1522.3 (+1.7%)	1478.5 (−1.2%)	1469.8 (−1.8%)
RCMSE	1.107	1.041 (−6.0%)	1.148 (+3.7%)	1.168 (+5.5%)
**Participant SC**				
%REC	12.04	13.25 (+10.0%)	12.16 (+1.0%)	10.06 (−16.4%)
MeanL	1515.8	1471.5 (−2.9%)	1503.3 (−0.8%)	1501.6 (−0.9%)
RCMSE	0.779	1.085 (+39.3%)	1.147 (+47.2%)	0.723 (−7.2%)

Note. Values represent session means. Values for V2–V4 are followed by percent changes from baseline in parentheses. %REC = percent recurrence; MeanL = mean diagonal line length; RCMSE = refined composite multi-scale sample entropy (scale 3). %DET (determinism) and %LAM (laminarity) were 100% across all participants and visits (ceiling effect) and were excluded from the interpretation. Higher RCMSE values indicate greater medium-timescale trunk acceleration complexity.

**Table 5:** Upper-extremity function and grip strength across timepoints.

Measure	Baseline	1-Month Post-SBC	3-Months Post-SBC
**Participant SA (Left hemiparesis)**			
UEFM	7	9	6
LEFM	12	16	11
BBT—Non-paretic (blocks/min)	38	43	52
BBT—Paretic (blocks/min)	0	0	0
Grip—Non-paretic (kg)	22.5	23.3	24.7
Grip—Paretic (kg)	2.9	1.0	1.0
**Participant SB (Left hemiparesis)**			
UEFM	36	43	37
LEFM	25	28	26
BBT—Non-paretic (blocks/min)	59	55	53
BBT—Paretic (blocks/min)	0	2	3
Grip—Non-paretic (kg)	25.3	25.0	22.8
Grip—Paretic (kg)	4.0	1.8	2.7
**Participant SC (Right hemiparesis)**			
UEFM	43	54	52
LEFM	29	29	29
BBT—Non-paretic (blocks/min)	61	69	59
BBT—Paretic (blocks/min)	6	10	7
Grip—Non-paretic (kg)	43.7	44.0	43.1
Grip—Paretic (kg)	12.1	14.5	11.1

Note. UEFM = Fugl-Meyer Assessment Upper Extremity (max 66); LEFM = Fugl-Meyer Assessment Lower Extremity (max 34); BBT = Box and Blocks Test (blocks per minute); Grip = dynamometer grip strength; SBC = Stroke Boot Camp. Assessments conducted at baseline (pre-SBC), 1-month post-SBC, and 3-months post-SBC.

**Table 6:** Mobility, balance, and functional outcome measures across timepoints.

Measure	Baseline	1-Month Post-SBC	3-Months Post-SBC
**Participant SA (Left hemiparesis)**			
10 MWT (m/s)	0.21	0.22	0.19
BERG	24	24	32
6 MWT (m)	67.6	57.0	68.0
FGA	0	6	10
SIS	56	54	51
**Participant SB (Left hemiparesis)**			
10 MWT (m/s)	1.35	1.17	1.21
BERG	51	53	56
6 MWT (m)	462.0	463.8	420.0
FGA	21	24	26
SIS	76	78	76
**Participant SC (Right hemiparesis)**			
10 MWT (m/s)	1.44	1.68	1.32
BERG	53	56	54
6 MWT (m)	328.8	444.8	420.0
FGA	29	30	28
SIS	73	75	75

Note. 10 MWT = 10-Meter Walk Test (mean of two trials, m/s); BERG = Berg Balance Scale (max 56); 6 MWT = 6-Minute Walk Test (meters); FGA = Functional Gait Assessment (max 30); SIS = Stroke Impact Scale (composite). Assessments were conducted at baseline, 1-month post-SBC, and 3-months post-SBC.

**Table 7:** Canadian Occupational Performance Measure (COPM) scores across timepoints.

	Baseline		1-Month Post-SBC		3-Months Post-SBC	
Goal	P	S	P	S	P	S
**Participant SA**						
Use two hands	2	3	4	3	2	1
Put on jacket	3	5	3	2	3	3
Walk smoother	4	5	6	7	4	5
**Participant SB**						
Grasp & release item	1	1	6	5	3	3
Walk faster w/o AFO	2	1	7	6	3	3
Open car door	2	1	7	6	3	2
**Participant SC**						
Swing arms while walking	1	1	4	5	6	8
Eat with utensils (R)	3	2	7	8	5	8
Computer mouse (R)	1	1	9	9	7	8

Note. AFO = Ankle–Foot Orthosis; P = performance; S = satisfaction (scale of 1–10; higher = better). Goals were self-identified at baseline. A change ≥2 points is considered clinically meaningful. Assessments were conducted at baseline, 1-month post-SBC, and 3-months post-SBC.

**Table 8:** APDM wearable sensor affected-to-unaffected limb symmetry ratios across four visits.

Sensor Measure	V1 (Baseline)	V2 (SBC Wk 1)	V3 (SBC Wk 2)	V4 (1-Mo F/U)
**Participant SA** (Left hemiparesis)				
Wrist acceleration A/UA	0.99	0.99	0.99	0.99
Wrist angular velocity A/UA	0.19	0.40	0.45	0.24
Foot acceleration A/UA	1.00	0.99	1.00	1.00
Foot angular velocity A/UA	0.62	0.84	1.31	0.76
**Participant SB** (Left hemiparesis)				
Wrist acceleration A/UA	0.99	1.00	0.99	0.99
Wrist angular velocity A/UA	0.61	0.58	0.46	0.62
Foot acceleration A/UA	0.98	0.99	1.00	0.99
Foot angular velocity A/UA	0.78	0.78	0.76	0.84
**Participant SC** (Right hemiparesis)				
Wrist acceleration A/UA	0.81	1.09	0.99	0.74
Wrist angular velocity A/UA	0.78	0.55	0.53	0.37
Foot acceleration A/UA	0.99	0.99	0.99	0.99
Foot angular velocity A/UA	0.88	0.80	0.76	0.82

Note. A/UA = affected-to-unaffected ratio; values approaching 1.00 indicate bilateral symmetry. Wrist sensors reflect upper-extremity kinematics; foot sensors reflect lower-extremity step dynamics. Acceleration = scalar resultant of three-axis acceleration (×10^6^ au); angular velocity = scalar resultant of three-axis gyroscope signal (×10^5^ au). V1 = baseline pre-SBC; V2 = SBC Week 1; V3 = SBC Week 2; V4 = 1-month follow-up.

## Data Availability

The data can be obtained from the author Dr. Alison McKenzie or the corresponding author. The Python codes used to analyze gait are available at FigShare; https://doi.org/10.60911/chapman.32561307.

## Abbreviations

The following abbreviations are used in this manuscript:

SBC	Stroke Boot Camp
FMA	Fugl-Meyer Assessment
BBS	Berg Balance Scale
FGA	Functional Gait Assessment
10MWT	10 m Walk Test
6MWT	6 min Walk Test
ApEn	Approximate Entropy
SampEn	Sample Entropy
RCMSE	Refined Composite Multi-Scale Entropy
IMU	Inertial Measurement Unit
GSD	Gait Sequence Detection
GSDA	Gait Sequence Detection A—Healthy Gait
GSDB	Gait Sequence Detection B—Pathological Gait
CWT	Continuous Wavelet Transform
HS	Heel Strike or Initial Contact
TO	Toe-Off or Final Contact
RQA	Recurrent Quantitative Analysis
SymEn	Symbolic Entropy
PermEn	Permutation Entropy
MSE	Multi-Scale Entropy
CMSE	Coarse Multi-Scale Entropy
SIS	Stroke Impact Scale
BBT	Box and Blocks Test
GD	Grip Dynamometry
GCT	Gait Cycle Time
SST	Single-Support Time
DST	Double-Support Time
LyE	Lyapunov Exponent
REC	% Recurrence
DET	% Determinism
LAM	% Laminarity
MeanL	Mean Diagonal Length
MaxL	Maximum Diagonal Length
EntrV	Vertical Line Entropy
EntrW	Waveform Entropy
MeanV	Mean Vertical Line Length
MaxV	Maximum Vertical Line Length
